# Particulars of a Case Resembling Syphilis, in Which the Uncertainty of Medical Opinions Is Strongly Exemplified

**Published:** 1814-12

**Authors:** 


### *For the Medical and Physical Journal.*

### *Particulars of a Case resembling Syphilis, in which the Uncertainty of Medical Opinions is strongly exemplified.* By W. O.

"Lectum tuum obsessum medicis scio, hinc prima mihi timendi causa est; discordant enim de industria, dum pudet novi nihil afferentem alterius hæsisse vestigiis, nec est dubium (ut eleganter ait Plinius,) omnes istos famam novitate aliqua aucupantes, Animas statim nostras negotiari, et in hac sola artium evenire ut cuicunque se medicum profitenti statim credatur, cum sit periculum in nullo mendacio majus. Nulla præterea lex quæ puniat inscitiam capitalem, nullum exemplum vindictæ: discunt periculis nostris, et experimenta per mortes agunt. Medico tantum hominem occidisse impunitas summa est, horum turbam velut inimicorum aciem, Clementissime Pater, intuere. Instruat te illius infausti epigrammatis memoria inscribi jubentis in sepulcro hoc solum 'TURBA MEDICORUM PERII.'"

*Petrarcha ad Clementem Sextum, P.M.*

ALTHOUGH it be very uncommon to find the excellent pages of the Medical Journal occupied with the productions of persons not of the medical profession, yet I hope I may be allowed to present a history of my own case, with such remarks as have struck me at various periods of my complaint, not only on the symptoms of the disease, but also on the various opinions I have had upon it from the most celebrated men of various countries; and, when it is considered that I have laboured under my affliction for several years, and instead of recovery have even become deplorably worse, I have a slight hope of turning the attention of the medical world to the consideration of similar cases; for I hope, that, without any degree of disrespect to your profession, I may acknowledge, that I have often been induced to upbraid its practitioners; as I believe I have experienced more of the uncertainty of their art than any man alive, inasmuch as I have even had opinions entirely contradictory as to the nature of my disease, and have been advised by one medical man to avoid, as certainly noxious or even destructive, what another has recommended to me as the only remedy for my complaint. I may also observe, that, during the series of years that I have been afflicted, I have, as far as books could assist me, endeavoured to form an opinion myself on the nature of my disorder, but so fluctuating has been the state of my mind among these contending authorities, that my opinion has even been as variable as that of my medical advisers.

About fifteen years ago, my father expressed a wish for my travelling on the Continent, previous to which time I had enjoyed an excellent state of health; and I may add, that our family has been remarkable for very long lives, and the very best health, for many ages.

A year after my departure from England I arrived at Paris,

Paris, (for, although the countries were at war, yet I was able, by particular circumstances, to travel over a considerable part of France,) where I had the misfortune to fall into great dissipation, the consequence of which was, beside the bad effects of excess in eating, drinking, and late hours, an attack of the venereal disease, in the form of chancres and bubos.

I applied to the first medical advice; M. Swediaur attended me; I took mercury in various forms, without its producing any effect upon my mouth; and, among other preparations of that medicine, they told me that in three months I had taken thirty-two grains of corrosive sublimate. My chancres healed; the bubos burst, became indolent, and for two or three years remained open, with callous edges, and sanious discharges; and now and then with slight attacks of inflammation, which was followed by an increased flow of matter, and comparative ease. By this time I had become considerably emaciated, but still continued, when sufficiently free from pain, my career of dissipation.

At length the ulcers in my groin healed, although my mouth had never been affected with mercury. I became then subject to pains of my bones, increased during the night; these particularly affected my right thigh; the limb swelled, the bone even could be felt enlarged, the disease extended from the hip almost to the knee; at length the part suppurated, and a small portion of black putrid matter discharged itself from one of the openings, for in a little time there were four places in the thigh from which purulent matter was discharged.

I now became uncommonly emaciated, my nights became miserable, the thigh-bone became more and more enlarged; I was obliged to take to my bed, and found it necessary to seek further advice. I left Paris, and went to London, without having any opinion as to the real nature of my disorder. I had much pain and swelling of my left knee, and sometimes of my elbows; I attributed it to rheumatism, or to the rheumatic gout, which has prevailed in some individuals of our family for many generations. The principal thing to be attended to, however, was the thigh. I made application to Sir ———, who affirmed that it was a case of necrosis, which opinion satisfied me for the time. I was advised to live well, and to trust more to the efforts of nature than to medical treatment; I therefore left London, and retired to my father's seat in ———, near which lived, secluded from the world, a surgeon who had been in extensive practice and great reputation. I requested this gentleman to give me his opinion as to the nature of my disorder; he seemed to

 agree agree with Sir ———, that the thigh was in a state of necrosis; and that nature might, after a great lapse of time, effect a cure: he stated also, that there was a probability of the complaint being *syphilitic*. I was thunder-struck; it had not entered into my mind, that the remains of that disorder still prevailed in my system; I had my complaint further considered, consultations were held, when it was determined that "there was no certainty of my complaint being syphilitic." The winter then was coming on; my body, worn out with sickness and pain, could not bear even the cold of September in England; I therefore determined to spend the winter months in the Mediterranean, for which I immediately departed. In the spring, (for pain and disease are querulous and greedy of change,) I went to ——— for further advice: my complaint had gained ground, the bone of my thigh was more enlarged than ever, I had swellings along the bones of my arms and shins, my knees were greatly enlarged, my limbs wasted to skin and bone, my appetite impaired, my nights miserably painful. I here received the advice of Mr. ———. Without giving me his opinion positively as to the nature of my disorder, he advised me to try what a little mercury would do: I took two or three of the blue pills, but they produced excessive griping and tenesmus, with profuse vomiting; I was obliged therefore to intermit this medicine.

I had long been accustomed to take large doses of laudanum at night, oftentimes a tea-spoonful or more, for my impatience would not allow me to number the drops. I fled to the Continent again, an object of misery, where I fell in with an ecclesiastic who commiserated my situation; he recommended me to take a kind of balsam, which he said would improve my appetite and strengthen me. I took one drop of it between breakfast and dinner, I believe it had great effect; my appetite sometimes became ravenous, I thought my strength improved, and I was in great hopes of having found a cure, when a branch of the English army arrived. I received further advice; the army physicians recommended me to keep up my strength and spirits, by good nourishment, and did not object to my continuing the medicine that the monk had recommended to me.

About this time I met an old acquaintance of mine, who said that he had for several years laboured under a distressing complaint that had baffled all the surgeons; that he had been obliged to retire to a warm climate; that he took the advice before leaving England of Sir ———, who recommended De Velnos' medicine, which had cured him. My friend advised me to take the same, and offered to furnish me with some that he had left. I refused his advice, however, though much importunity was made use of, as I had heard that that medicine was only a preparation of mercury, so that I concluded that Lord ——— had laboured under some irregular syphilitic disorder; for at this time I had satisfied myself, from the uncertainty of the medical opinions I had received, and the positive assertions of some surgeons of note, that my complaint was not of a syphilitic nature.

About this time I had another alarming symptom; my servant found me one day lying on the floor insensible, with my eyes fixed, and my face black, from which attack I soon recovered, but had several other similar paroxysms, during the succeeding months. I could never from my medical attendants procure a positive opinion on the nature of these attacks; one physician said, that they were occasioned by the large quantities of opium I took over night; another, that they depended on irritation about the intestines, (for I was at this time much affected with spasms of these parts;) a third, that they arose from a disease of the head: whatever the cause might have been, however, I determined to proceed to England immediately, and spend the summer there. I accordingly took ship for a port whence there is often a conveyance to England. This port was a great naval depot; there was a surgeon there who was spoken of as a man of great knowledge in his profession, and I was advised to consult him.

At this time, beside my left thigh being in *statu quo*, there were tumours appearing and disappearing upon my shin bones and arms, soft tumours to the feel, containing a fluid, appeared about my forehead; one of the orifices of the left thigh discharged a thin matter; I was excessively emaciated, so weak that I scarcely could walk, and my nights were passed with great uneasiness; I was often subject to great pain about the stomach, and my appetite was very bad. I took this gentleman's advice, who gave me a positive opinion as to the nature of my case, which I was glad to find was the same as my own, and I was the more gratified on receiving a positive opinion, as most others of my medical advisers had given a doubtful one, and left me in an uncomfortable uncertainty.

This gentleman told me that my case was certainly not *venereal*, but occasioned by the *mercury* that I had taken; he advised me, therefore, by all means never again to touch a grain of mercury, as I valued my life; and that I might attribute to that medicine all my complaints. I therefore continued still to take my stomachic medicine, and trusted to good living for my recovery.

I remained

I remained under this gentleman's treatment about a month with little change of symptoms, when I was fortunate enough to procure a passage in a ship of war to England; but judge my astonishment, on my putting myself under the treatment of the surgeon of the ship, who altogether controverted the opinion of the former surgeon, and was as positive the contrary way. He told me that he considered most of the symptoms under which I laboured as syphilitic; that the fits of insensibility under which I laboured were probably epileptic, occasioned by accidental irritation, that the affection of the thigh had probably a syphilitic origin, that the swellings of the bones of the arms and legs were venereal nodes, the tumours about my forehead also venereal, that I might also be subject to gouty or rheumatic affections, and that the large doses of laudanum impaired my appetite and weakened my digestion; and he even doubted as to the reality of my thigh-bone being in a state of necrosis, asking me whether there had been any fragments of bone discharged, whether a probe had been introduced, and the dead bone felt, and whether there was a sort of fleshy tumour about the orifice. To these questions I replied, when the discharge first took place, the matter was fetid and black; I thought it osseous or dissolved bone, but that there were no particles of bone mixed with it, so that I was not positive whether it really consisted of bony matter or not. I gave an answer to the second question, by permitting the surgeon to inspect the orifices, who found that there was *no* fleshy tumour about the openings. I had not heard of this appearance being a common indication of necrosis before, and I began myself to be doubtful on that head, although the surgeon did not deny that there might be necrosis present; yet he affirmed, that the disease of the thigh-bone might be similar to those of the legs and arms, but in a more advanced state. What could I do? I was in a greater degree of uncertainty than ever; I had received two opinions directly opposite to each other, one attributing to mercury all my sufferings, the other as positively presenting that medicine to me as the only cure for my disorder! How could I determine the true state of the case among such jarring opinions? How could I fix upon the most intelligent of these two surgeons, both of them holding public situations, and both looked up to as proficients in their professions? I could only from the opinions they gave me endeavour to elicit the truth; I shall describe, therefore, what each said, in proof of the validity of his opinion: and I cannot here fail from observing, that to many the subject may seem to tend to ridicule, and bring to their recollections the contest between the doctors in Gil Blas, and other other books, where similar opprobria to the profession are made use of. When they call to mind, however, that *here* was no meeting between the two surgeons, and no dispute in the presence of their patient, I hope they will have a different view of the matter; and I entreat that they will have some commisseration for my sufferings, and with me agree, that it would certainly be of use to other sufferers labouring under the same symptoms, to determine accurately on the subject, and not attribute symptoms to one cause which are produced specifically by another.

I recollect well, that, when I conversed with the gentleman who determined my case to be mercurial, and who recommended me, as I valued my life, never to swallow a particle of mercury again, he gave me an account of what he had met with in the course of his practice, which induced him to form the opinion he now gave me, and which, he said, since that time, had been further corroborated.

"I formerly (said he) was ordered to take charge of the patients in a large hospital, the former surgeon having died. In that hospital I found near thirty miserable objects labouring under what had been called venereal ulcers, and other syphilitic affections; some of these appeared to be at the last gasp of life, being wasted to the bone, with large ulcers in the groins, legs, throats, heads, &c. and many of them suffering the most grievous pains. On a reference to the treatment they had been put under by the former surgeon, I found that they had uniformly been taking corrosive sublimate, some of them for several months, during which time, instead of their sores healing, and their pains diminishing, they became worse, and many of them were as much reduced as you are. The idea then struck me, that these patients, instead of labouring under syphilis, were suffering through the effects of mercury. I therefore laid this medicine entirely aside, prescribed a better food for them, and gave them tonic medicines; in a little time their ulcers put on a better appearance, and in two or three months their cures were complete. I see but very little difference between some of these cases and your's: *they* had ulcers, so have *you; they* had swellings and pains of the bones, so have *you; they* had taken much corrosive sublimate, so have *you; they* were greatly emaciated, so are *you*. I would strongly recommend to you, therefore, to preserve a generous diet, use as much exercise as your health will admit, and avoid mercury as your bane, by which means I have hardly a doubt, but that in time you will have as perfect a cure as the patients I have just mentioned to you."

Such an opinion could not fail of being very gratifying to me; it was also consonant to my own; it enlivened my spirits, and I confidently expected, by an adherence to it, that I should in the end perfectly recover my health.

So far for my antisyphilitic adviser,—now for my syphilitic one. This gentleman, before giving me his ideas on the subject, seemed to wish me to inform him what had been the opinions of the medical men I formerly had consulted, and I must confess that I was a little disingenuous with him on that head. We were perfect strangers to each other, and I wished to have his opinion unbiased by that of others. I however told him the purport of the last advice I had received, which was necessary to account for the treatment I was then employing, and it was indeed that which was more agreeable to myself; I also mentioned that there was no supposition of my complaint being venereal; that I had consulted Sir ——, Dr. ——, Dr. ——, Dr. ——, Dr. ——, Mr. ——, Mr. ——, Mr. ——, &c. none of whom had given an opinion of that kind. He seemed to be struck with my answers, and I thought he seemed to wish to give a contrary opinion, but that from delicacy or diffidence was unwilling to controvert these high authorities.

He demanded of me, whether, when I had consulted Sir ——, I had the swellings of the shins and arms, the soft tumours about the head, &c. which I now laboured under: and on my answering in the negative, he said, that he believed, if Sir —— saw me now, he would have no doubt as to the nature of the complaint, in opposition to the ideas of the other gentlemen I had mentioned. He then gave me the following as his firm opinion: "The swellings about your head are venereal; the irregularities on the arms and shin-bones are venereal exostoses; the pains you suffer in the night are the usual nocturnal pains of that disorder; the disease of the thigh-bone, whether it be really necrosis or not, has probably a like origin; you may perhaps also be subject to rheumatism or gout, mercury often produces the former, and the latter is hereditary in your family. Although you may have taken much mercury, as you affirm that your mouth was very little affected by it, it is probable you never took a sufficient quantity to cure the disorder; for I have great doubts whether mercury will eradicate a confirmed lues, without producing some degree of ptyalism, however much may be taken. The tale told you by the surgeon of the hospital of the patients who had taken large quantities of corrosive sublimate without being cured, proves nothing in favour of your complaint not being syphilitic; the men at that place had probably taken enough mercury, or perhaps too much, to eradicate the syphilitic affections that they had at first laboured boured under, and their ulcers then were of the nature of common indolent ulcers, the body being too weak to effect a cure; whence on a more generous diet being prescribed, nature was invigorated, and the men recovered. I would also controvert the opinion of your disorder not being syphilitic, by saying that the effects of mercury on the system are very well known. However extensively taken, mercury does *not* produce nodes on the middle of cylindrical bones, or on the cranium; the principal deleterious effects of the long-continued exhibition of mercury, are rheumatic pains; I do not know that mercury ever has been known to produce tumours on the cylindrical bones when given to patients not labouring under syphilitic affections. Why do not those persons who suffer the most violent and repeated salivations for liver complaints, fall into disorders similar to your's, if mercury had, or possibly could have, the power of producing exostoses on the head, tibiæ, or ulnæ? I believe such effects have never been heard of as consequent to such disorders, or such a remedy. I would therefore advise you strongly to return to the use of mercury; as you are very weak, take it in small quantities, use a generous diet; for the cure of your thigh-bone trust much to the efforts of nature; when you arrive in England, take the Lisbon diet-drink in conjunction with mercury, and take further medical advice."

Here I am, therefore, still in a state of uncertainty. Whom am I to believe? Am I to look upon mercury as my bane or my saviour? Unwillingness to suppose my body destroyed by the poison of syphilis, induces me to give credit to the former opinion; and the being enabled to make use of a specific and certain remedy, and thereby receive a perfect cure, tends much to turn me to the latter.

*London, August* 1814. W. O.

---

*For the Medical and Physical Journal.*

